# High-Throughput mRNA Sequencing Reveals Potential Therapeutic Targets of Febuxostat in Secondary Injury After Intracerebral Hemorrhage

**DOI:** 10.3389/fphar.2022.833805

**Published:** 2022-06-23

**Authors:** Xueyan Wang, Chenyu Zhang, Yuwen Li, Ting Xu, Jin Xiang, Yang Bai, Ying Zhang, Qi Wang, Tiejun Zhang, Linchuan Liao

**Affiliations:** ^1^ Department of Pharmacy, West China Hospital, West China School of Basic Medical Sciences & Forensic Medicine, Sichuan University, Chengdu, China; ^2^ Department of Pharmacy, West China Hospital, Sichuan University, Chengdu, China; ^3^ West China School of Pharmacy, Sichuan University, Chengdu, China; ^4^ Clinical Trial Center, West China Hospital, Sichuan University, Chengdu, China; ^5^ Department of Neurosurgery, West China Hospital, Sichuan University, Chengdu, China; ^6^ Department of Forensic Analytical Toxicology, West China School of Basic Medical Sciences & Forensic Medicine, Sichuan University, Chengdu, China

**Keywords:** febuxostat, intracerebral hemorrhage, neuroprotection, high-throughput mRNA sequencing, Wisp1

## Abstract

Febuxostat is a urate-lowering medication for the treatment of patients with gout. This study was performed to elucidate the effects and underlying mechanisms of febuxostat on neuronal injury induced by intracerebral hemorrhage (ICH) in mice. The results showed that the administration of febuxostat improved neurological severity scores and blood–brain barrier (BBB) permeability. Moreover, febuxostat attenuated neuronal cell death and cytokine levels compared with the ICH group. Next, we conducted a transcriptome analysis of the neuroprotective effects of febuxostat. The overlapping significant differentially expressed genes (DEGs) were identified. Gene ontology (GO) analysis revealed that the overlapping significant DEGs were most enriched in five items. The intersecting DEGs of the aforementioned five pathways were Wisp1, Wnt7b, Frzb, and Pitx2. In addition, GO terms and Kyoto Encyclopedia of Genes and Genomes (KEGG) pathways revealed that DEGs were mainly involved in the wnt signaling pathway. Furthermore, the expression of Wisp1 and Wnt7b in the perihematomal region at 72 h post-ICH was observed. The results showed that both Wisp1 and Wnt7b were increased in the ICH group and were decreased by the administration of febuxostat. Taken together, the study showed that febuxostat protected against secondary brain injury after ICH and the Wnt7b-Wisp1 pathway was closely related to neuroprotective effects.

## 1 Introduction

Febuxostat is an orally administered, nonpurine-selective xanthine oxidase inhibitor approved by the Food and Drug Administration (FDA) in 2009 for the management of hyperuricemia in patients with gout (Hair et al., 2008; Kamel et al., 2017; Robinson and Dalbeth, 2018). In addition to the treatment of gout, febuxostat played roles in preventing the progression of periodontitis (Nessa et al., 2021), preventing the cytotoxicity of propofol in brain endothelial cells (Hao et al., 2021), attenuating testosterone-induced benign prostatic hyperplasia (Abdel-Aziz et al., 2020), ameliorating methotrexate-induced lung damage (Zaki et al., 2021), and mitigating concanavalin A-induced acute liver injury (Maresh et al., 2020) in animal experiments. Moreover, febuxostat suppressed renal ischemia-reperfusion injury by modulating oxidative stress and promoting the resynthesis of adenine nucleotides (Tsuda et al., 2012; Fujii et al., 2019). Febuxostat also improved cardiac ischemia-reperfusion injury and inhibited inflammation in response to acid-induced acute lung injury and acetaminophen liver injury (Kataoka et al., 2015; Wang et al., 2015; Fujii et al., 2019).

Intracerebral hemorrhage (ICH) is the second most common subtype of stroke after ischemic stroke, comprising 10%–15% of all strokes, and is associated with greater morbidity and mortality than ischemic strokes (Ikram et al., 2012; An et al., 2017; Ziai and Carhuapoma, 2018; Garg and Biller, 2019). ICH induces brain damage due to an initial pathological disruption from mechanical compression caused by hematoma, and subsequently, brain hernia and brain edema cause secondary brain injury, which is related to inflammation, oxidative stress, cytotoxicity of erythrocyte lysates, and neurotoxicity of thrombin (Wu et al., 2016; An et al., 2017; Shao et al., 2019). Global transcriptome profiling using RNA sequencing in the peripheral blood of patients at 72 h after ICH revealed DEGs that were highly expressed, including those related to inflammation and activation of the immune response (Walsh et al., 2019). Preventing secondary ICH injury using antithrombotics and statins in patients has been a major treatment target and research topic in recent years. Regrettably, the role of the aforementioned drug therapy in ICH survivors remains controversial and the clinical outcome remains unsatisfactory (Hostettler et al., 2019). This study was performed to elucidate the effects and underlying mechanisms of febuxostat on neuronal injury induced by intracerebral hemorrhage (ICH) in mice.

## 2 Materials and Methods

### 2.1 Animals

The animal protocol was approved by the Committee on the Ethics of Animal Experiments of West China Hospital of Sichuan University (Approval protocol number: 20211480A). Male C57BL/6 mice (weight 20–25 g) were obtained from Chengdu DOSSY Experimental Animals Co. Ltd. (Sichuan, China). Animals were housed under controlled laboratory conditions (12 h light/dark cycle, controlled temperature and humidity, free access to standard food and water, and minimum-range environmental noise). All efforts were made to minimize any potential suffering and the number of animals used. All experimental procedures and outcome assessments were performed in a blinded manner.

### 2.2 Drugs and Reagents

Febuxostat was purchased from Tejin Pharma Limited (Tokyo, Japan, 20 mg, Lot. No. 6474) and suspended in ultrapure water. Chloral hydrate was purchased from West China Hospital of Sichuan University (Sichuan, China). The TRIzol reagent and Wisp1 polyclonal antibody were obtained from Invitrogen; Thermo Fisher Scientific, Inc. (Waltham, MA, United States). The Wnt7a/b antibody was obtained from Santa Cruz Biotechnology (Santa Cruz, CA).

### 2.3 Intracerebral Hemorrhage Model and Experimental Design

The mice were anesthetized intraperitoneally with chloral hydrate (Zhao et al., 2017; Wang et al., 2018). The mice were positioned in a mouse stereotaxic frame and 30 μl of autologous whole blood was injected at a rate of 2 μl/min with a micro-infusion pump. The injection coordinates were 0.3 mm anterior and 2.2 mm lateral from the bregma at the skull surface at a depth of 3.3 mm. The needle was left in place for 10 min and then slowly pulled out. C57BL/6 mice (20–25 g) were randomly assigned to the following three groups: 1) sham treatment group (sham, injected with saline), 2) intracerebral hemorrhage model group (ICH, injected with autologous blood), and 3) febuxostat group (feb) in which the mice were treated with febuxostat suspension (50 mg/kg) once daily for 5 consecutive days by gavage before intracerebral hemorrhage modeling.

### 2.4 Behavior Tests

The behavior tests included the modified neurological severity score, the wire hanging test, the beam walking test, and the forelimb placing test, which were described previously (Chen et al., 2001; Seyfried et al., 2009; Luong et al., 2011; Matsushita et al., 2011; Krafft et al., 2014; Xu et al., 2017; Wei et al., 2021). Before the behavior tests, all animals underwent behavior training for 3 days, and abnormal animals were excluded. All behavior tests were performed and evaluated by two trained investigators who were blinded to the animal groupings.

### 2.5 Modified Neurological Severity Score

The modified neurological severity score (mNSS) was used, which consisted of motor tests, sensory tests, beam balance tests, reflex absence, and abnormal movements. The neurological deficits of the mice 24 h after ICH were evaluated. The higher the score, the greater the degree of neurological injury (normal score = 0; maximal deficit score = 18) will be.

### 2.6 Wire Hanging Test

The wire hanging apparatus was a stainless steel wire (50 cm length, 2 mm diameter) resting on two vertical supports and elevated 40 cm above a flat surface. The mice were placed on the middle of the wire with the two forepaws and were observed for 30 s in three trials. The mice were scored according to the following criteria: 0, fell off; 1, hung onto the wire with two forepaws; 2, hung onto the wire with added attempts to climb onto the wire; 3, hung onto the wire with two forepaws and one or both hind paws; 4, hung onto the wire with all four paws and with the tail wrapped around the wire; and 5, escaped to one of the supports.

### 2.7 Beam Walking

The test was performed to evaluate fine motor coordination and function by measuring the ability of the animals to traverse an elevated narrow beam. The mouse was placed on a beam (1.2 m long, 1.0 cm wide, and 40 cm high). The time for the mouse to traverse the beam (not to exceed 60 s) and the hindlimb performance as it crossed the beam (based on the 1 to 7 rating scale) were recorded according to the following criteria: 0, could not balance on the beam (<5 s); 1, remained on the beam for >5 s but could not cross the beam; 2, could balance on the beam but could not traverse it; 3, traversed the beam with the affected limb extended and did not reach the surface of the beam or made a turn on the beam; 4, traversed the beam with 100% foot slips; 5, traversed the beam with >50% but <100% foot slips; 6, traversed the beam with <50% foot slips; and 7, traversed the beam with two or fewer foot slips.

### 2.8 Forelimb Placing Test

The ability of the mouse to respond to a vibrissae-elicited excitation by the forward movement of its forelimb was evaluated with the forelimb placing test. The mouse was held by the trunk in parallel to a tabletop and then moved slowly and vertically until the vibrissae on one side touched the table surface. Refractory placements of the impaired (left) forelimb were evaluated, and a score was calculated as the number of successful forelimb placements out of 10 consecutive trials. Forward movements of the ipsilateral paw were recorded out of 10 repeated trials.

### 2.9 Histopathology Analysis

#### 2.9.1 Brain Section

The mice were anesthetized with inhaled isoflurane. After the brain was perfused for 5 min through the left cardiac ventricle with 0.9% NaCl injection (0.9%, Chengdu Qingshan Li Kang Pharmaceutical Co. Ltd., Sichuan, China) until the effluent from the right atrium was clear, the brains were removed, postfixed in 4% paraformaldehyde overnight, and then sectioned coronally (10 μm) over the entire region of injury.

### 2.10 Fluoro-Jade B Staining

The brain sections were placed in a 0.06% potassium permanganate solution and subsequently incubated with 0.001% FJB solution. Finally, the brain sections were observed under a fluorescence microscope (Nikon, Japan).

### 2.11 Terminal Deoxynucleotidyl Transferase dUTP Nick End Labeling Staining

According to the manufacturer’s instructions (TUNEL assay kit, Servicebio, China), TUNEL staining was performed to detect cellular apoptosis in the brain tissues around the hematoma from all of the groups. The TUNEL-positive cells in the brain tissues around the hematoma were observed and analyzed by ortho-fluorescence microscopy (Nikon, Japan).

### 2.12 Cytokine ELISA Assay

At 72 h after ICH, supernatant samples from the perihematomal brain tissue homogenate were obtained. IL-1β, IL-6, and IL-18 levels in brain tissues were determined by using ELISA kits (Abcam, United States) according to the protocols provided by the manufacturers.

### 2.13 Evaluation of BBB Permeability

To evaluate BBB permeability, 4% Evans blue (EB) (Solarbio, China) was injected intraperitoneally at 72 h post-ICH induction and circulated *in vivo* for 4 h. Then, the mice were transcardially perfused with saline. Subsequently, the brains were removed and fixed with paraformaldehyde. In addition, for the identification of vascular structures within the hematoma region, immunofluorescence was performed for the endothelial cell marker CD31. The brains were paraffin-embedded and cut into 4 mm thick sections for co-immunofluorescence of EB and CD31.

### 2.14 RNA Extraction, Library Preparation, and Sequencing

Total RNA was extracted from brain tissue using the TRIzol reagent (Invitrogen) according to the methods of Chomczynski et al. (DOI:10.1006/abio.1987.9999). DNA digestion was carried out after RNA extraction by using DNaseI. To prepare the library for RNA-seq, a Ribo-off rRNA Depletion Kit (Catalog NO. MRZG12324, Illumina) and KC-DigitalTM Stranded mRNA Library Prep Kit for Illumina^®^ (Catalog NO. DR08502, Wuhan Seqhealth Co., Ltd. China) were used according to the manufacturer’s instructions. The kit eliminates duplication bias in the PCR and sequencing steps by using a unique molecular identifier (UMI) of eight random bases to label the preamplified cDNA molecules. The library products corresponding to 200–500 bps were enriched, quantified, and finally sequenced on a NovaSeq 6000 sequencer (Illumina) with the PE150 model.

### 2.15 RNA Seq Data and Bioinformatics Analysis

Raw sequencing data were first filtered by Trimmomatic (version 0.36), low-quality reads were discarded, and the reads contaminated with adapter sequences were trimmed. Clean reads were first clustered according to the UMI sequences, in which reads with the same UMI sequence were grouped into the same cluster. Reads in the same cluster were compared to each other by pairwise alignment, and then reads with a sequence identity of more than 95% were extracted to a new subcluster. After all subclusters were generated, multiple sequence alignment was performed to obtain one consensus sequence for each subcluster. RNA-seq reads were aligned to the Ensembl mouse genome built GRCm38 using STRA software (version 2.5.3a) with default parameters. Reads mapped to the exon regions of each gene were counted by feature counts (Subread-1.5.1; Bioconductor), and RPKM was calculated. Genes differentially expressed between groups were identified using the edgeR package (version 3.12.1). Significant differentially expressed genes were identified as such if the fold change was larger than 2.0 and the *p-*value was <0.05. Enriched Gene Ontology (GO) for annotated differentially expressed genes and enriched Kyoto Encyclopedia of Genes and Genomes (KEGG) pathways were performed by KOBAS software (version: 2.1.1) with a *p-*value cutoff of 0.05 to judge statistically significant enrichment.

### 2.16 Protein–Protein Interaction Network Construction

STRING 11.0 (https://www.string-db.org/) was used to explore protein–protein interaction (PPI) networks and functional relations in differentially expressed proteins (DEPs) based on interactions with combined scores ≥0.15. Cytoscape (version 3.7.2) was used to visualize the network.

### 2.17 Immunohistochemical Staining

The paraffin-embedded sections were deparaffinized, rehydrated, and subjected to antigen retrieval, followed by treatment with 3% hydrogen peroxide to block endogenous peroxidase activity. After serum sealing, the sections were incubated with anti-Wisp1 (1:100) or anti-Wnt7a/b (1:200) overnight at 4°C and then covered with a secondary antibody from the corresponding species of the primary antibody and incubated at room temperature for 50 min. Then, the sections were treated with chromogenic DAB, counterstained with hematoxylin stain solution, dehydrated, and mounted. The sections were observed under a microscope.

### 2.18 Immunofluorescence Staining

To assess the protein expression of Wisp1 or Wnt7b, immunofluorescence labeling was performed by incubating sections with anti-Wisp1 (1:100) or anti-Wnt7a/b (1:200) overnight at 4°C. The sections were washed, followed by incubation with FITC-labeled IgG (Servicebio, Hubei, China). DAPI was used to stain the nucleus. The sections were observed under a fluorescence microscope.

### 2.19 Statistical Analysis

Statistical analysis was performed using GraphPad Prism software (version 9.10). Comparisons between the groups were carried out using the *t*-test or analysis of variance. *P-*values < 0.05 were considered to be statistically significant.

## 3 Results

### 3.1 Febuxostat Improved Neurological Dysfunction After ICH

**FIGURE 1:**
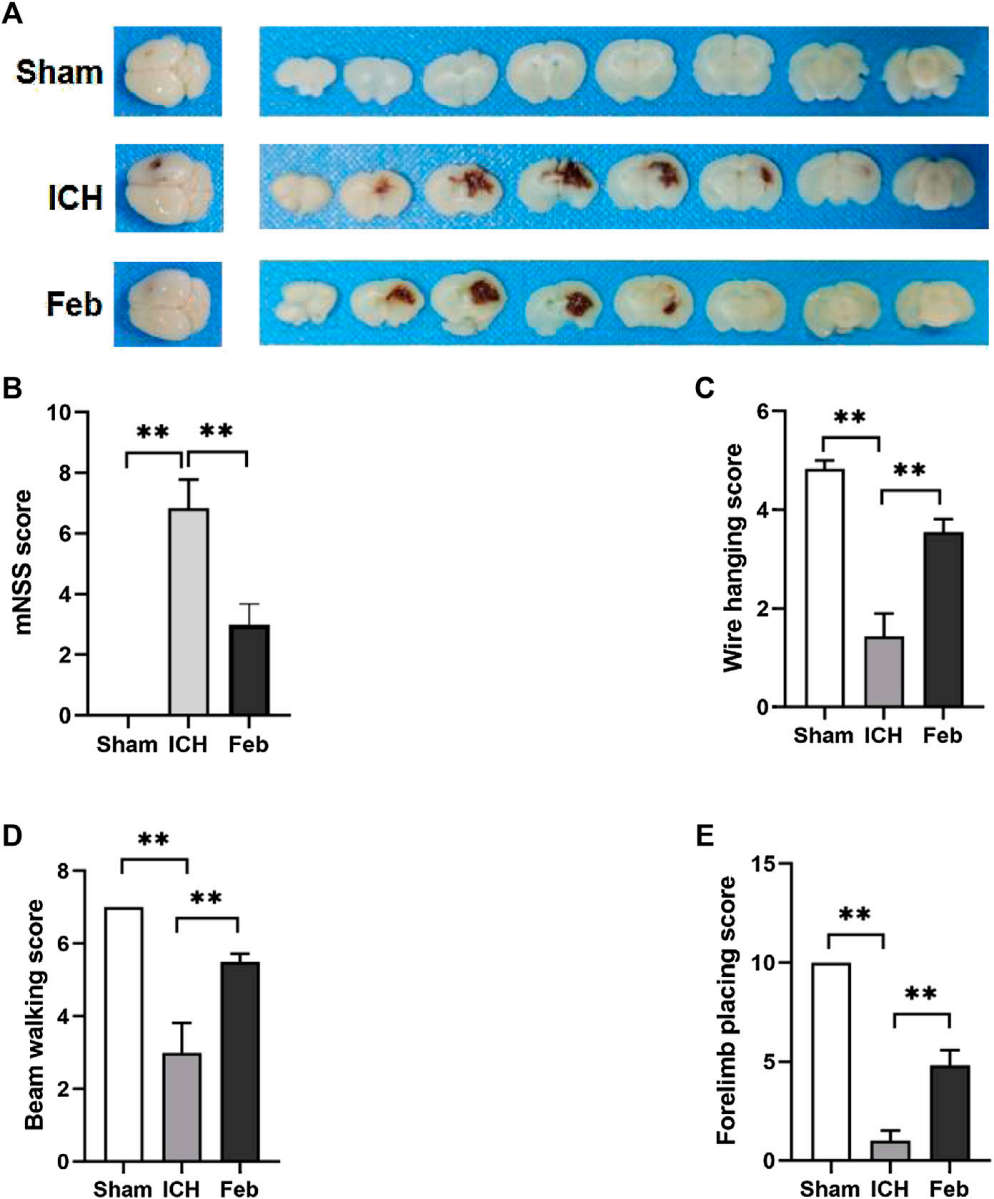
Febuxostat improved neurological dysfunction after ICH: **(A)** Representative images of coronal brain sections; **(B)** Modified neurological severity score. **(C)** Wire hanging test. **(D)** Beam walking test. **(E)** Forelimb placing test. Values are mean ± S.E.M; **p < 0.01 (*n* = 6 mice/group, two-way ANOVA).

An ICH model with autologous blood was constructed, and a schematic representation of the coronal brain sections is shown in Figure 1A. The mNSS was used to assess neurological deficits. The wire hanging test, beam walking test, and forelimb placing test were performed to evaluate motor and coordination functions. ICH-induced neurological deficits were significantly alleviated by the administration of febuxostat (Figure 1B). In addition, febuxostat increased the latency to fall in the wire hanging test and beam walking test and improved forelimb placing behavior compared with the ICH group. Thus, febuxostat treatment improved locomotor abnormalities and sensorimotor deficits after ICH (Figures 1C–E).

### 3.2 Febuxostat Attenuated Neurodegeneration and Apoptosis After ICH

**FIGURE 2:**
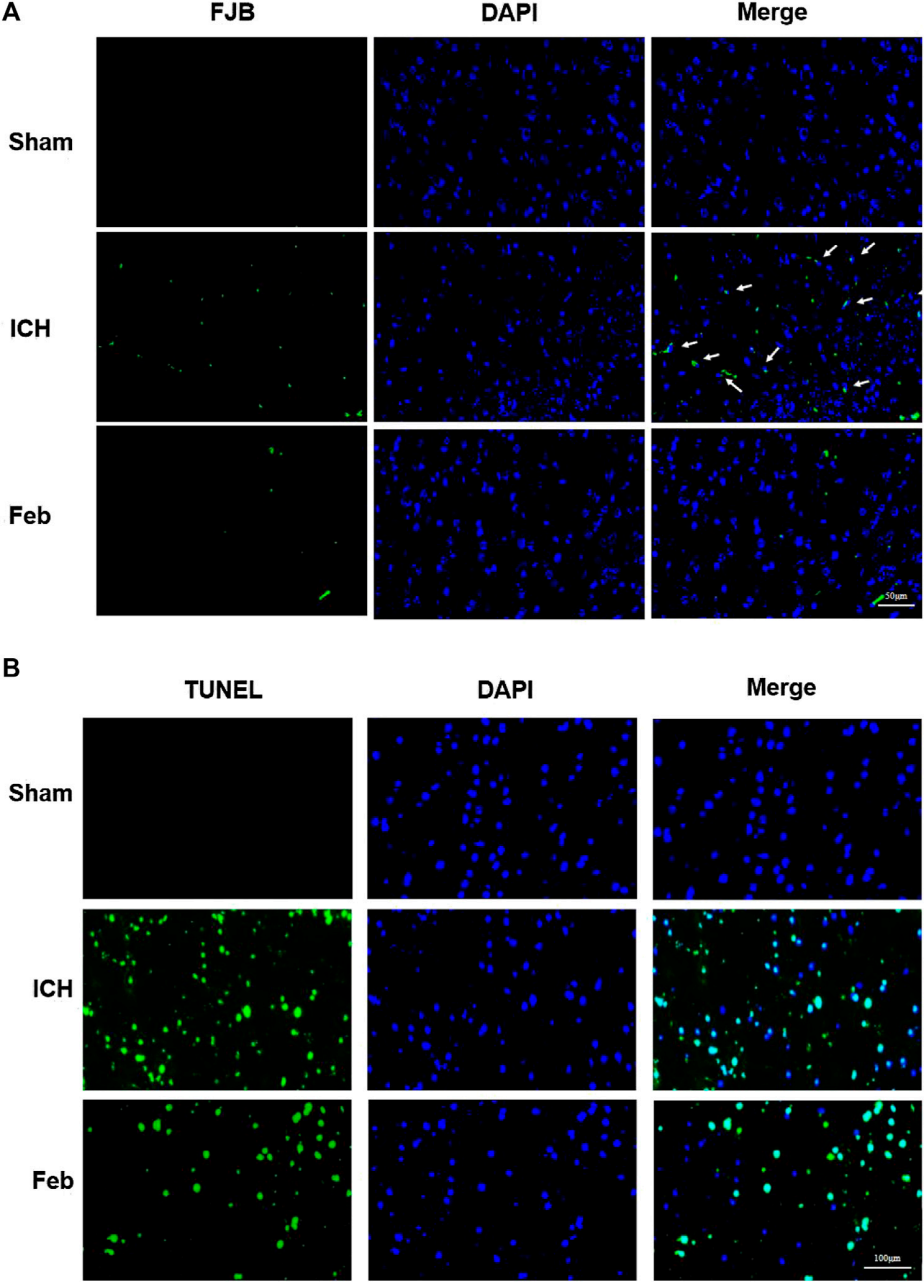
Febuxostat attenuated neurodegeneration after ICH: **(A)** Degenerating neurons in the brain sections were labeled with FJB stain. Neuronal degradation was stained green. White arrows indicate colocalization. The scale bar is 50 μm. **(B)** Apoptotic neurons in the brain sections were labeled with the TUNEL stain. TUNEL-positive cells are shown in green. The scale bar is 100 μm. Sections were stained with DAPI (blue) to show all nuclei.

To evaluate neuronal degeneration and apoptosis in the perihematomal region at 72 h post-ICH, the brain sections were subjected to FJB staining (Figure 2A) and TUNEL staining (Figure 2B). The number of FJB-positive and TUNEL-positive cells was significantly increased after ICH but was remarkably decreased by febuxostat treatment, suggesting that febuxostat could alleviate brain injury after ICH.

### 3.3 Febuxostat Decreased the Expression of Proinflammatory Cytokines After ICH

The release of inflammatory cytokines after ICH was measured by an ELISA kit. As shown in Table 1, the levels of IL-1β, IL-6, and IL-18 in perihematomal tissue after ICH were obviously increased compared with those in the sham group. The administration of febuxostat decreased the levels of inflammatory cytokines compared with the ICH group.

**TABLE 1:** IL-1β, IL-6, and IL-18 levels in the sham, ICH, and feb groups.

Group	n	IL-1β (pg/mg)	IL-6 (pg/mg)	IL-18 (pg/mg)
Sham	6	42.16 ± 1.22	61.76 ± 1.34	25.24 ± 1.28
ICH	6	160.72 ± 2.51	201.28 ± 1.86	178.84 ± 3.12
Feb	6	84.32 ± 2.30^a^	125.52 ± 3.24^a^	104.56 ± 2.83^a^

a Compared with the ICH model group, *p*< 0.05.

### 3.4 Febuxostat Improved BBB Integrity After ICH

**FIGURE 3:**
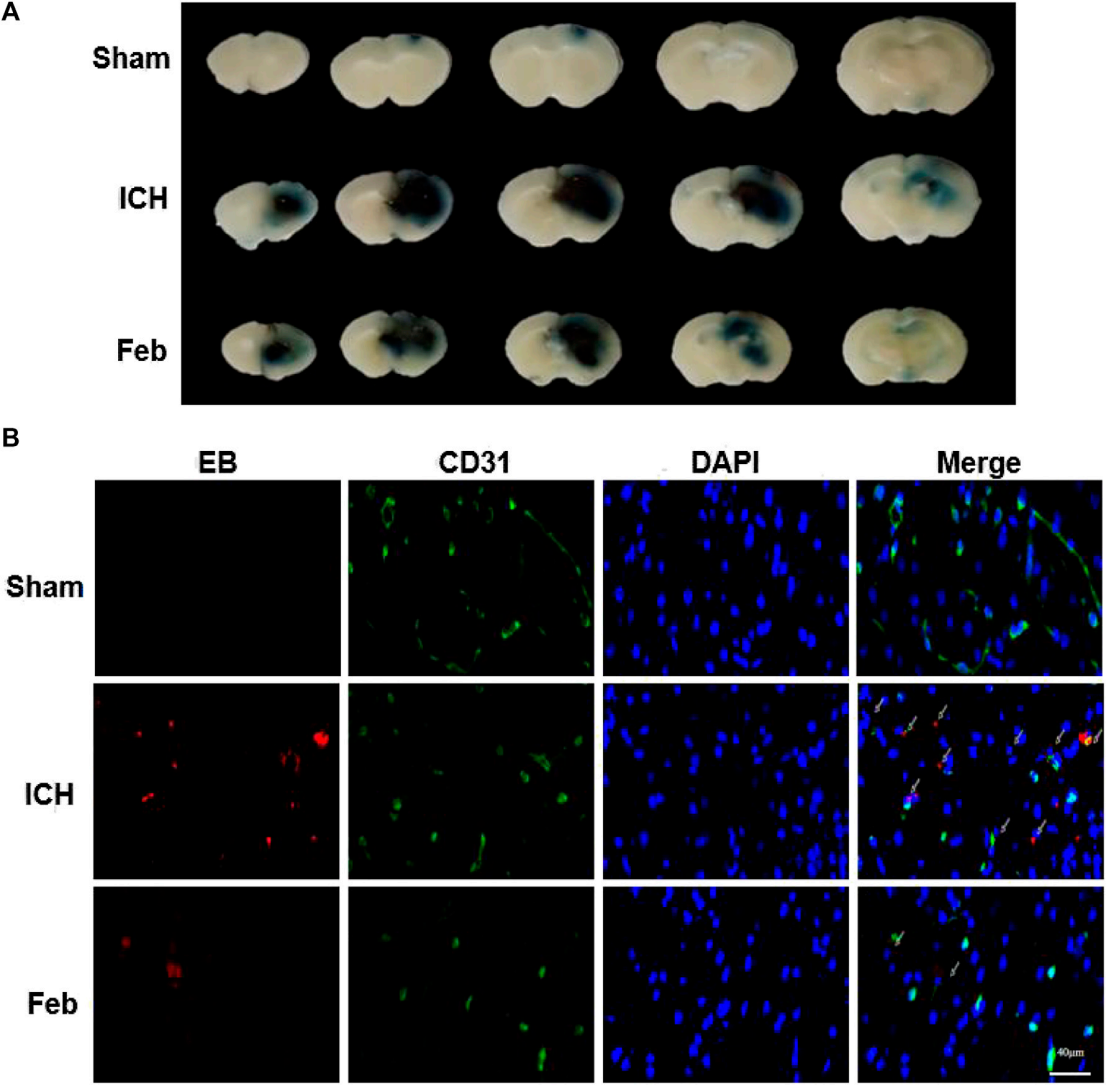
Febuxostat attenuated BBB permeability after ICH: **(A)** Representative coronal brain sections show Evans blue extravasation. **(B)** Permeability of the BBB using the Evans Blue (EB) assay. EB is shown in red. CD31 is shown in green. White arrows indicate colocalization. Sections were stained with DAPI (blue) to show all nuclei. The scale bar is 40 μm.

The integrity of the BBB was evaluated by co-labeling EB with the vessel marker CD31, an angiogenesis-related protein, in coronal brain slices. As shown in Figures 3A,B, EB extravasation was reduced in the group treated with febuxostat compared with the ICH group, indicating that febuxostat protected against BBB disruption after ICH.

### 3.5 Analysis of Differentially Expressed Genes

**FIGURE 4:**
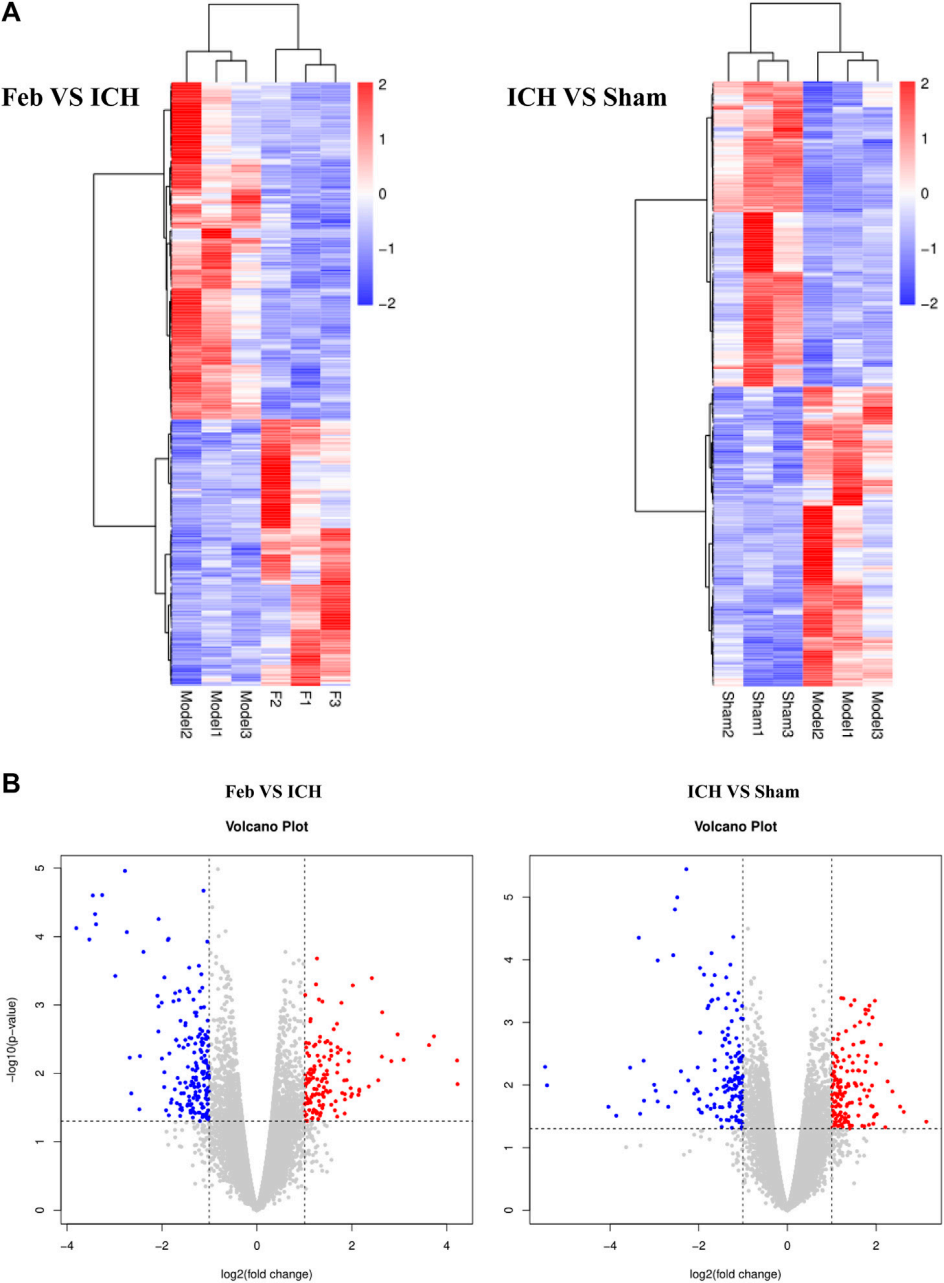
Hierarchical cluster analysis of DEGs: **(A)** Hierarchical cluster analysis showed that the DEGs ultimately clustered into two major branches: upregulated genes (labeled in red) and downregulated genes (labeled in blue). **(B)** Volcano plot of DEGs. The red and blue points in the plots represent the significantly upregulated and downregulated DEGs. *n* = 3 mice/group.

To investigate the molecular mechanisms of febuxostat in neuroprotection, gene expression profiling was performed. According to the filtering criteria *p* < 0.05 and fold change >2, 322 differentially expressed genes were identified between the feb and ICH groups, of which 142 were upregulated and 180 were downregulated. Subsequently, 154 genes were significantly downregulated and 151 genes were significantly upregulated in the ICH group compared with the sham group. Distinct expression patterns were observed by hierarchical clustering analysis and a volcano plot (Figure 4).

In addition, overlapping significant DEGs were identified from the intersection of DEGs (ICH group vs. sham group) and DEGs (feb group vs. ICH group). The list of the top five known down- and upregulated DEGs, ranked by log2 (fold change), is shown in Table 2.

**TABLE 2:** Top five DEGs with log2 (fold change) among the overlapping significant DEGs.

Down-DEGs			Up-DEGs		
NO.	Symbol	logFC	NO.	Symbol	logFC
1	Abi3bp	−2.6480	1	Pitx2	3.7292
2	Wisp1	−2.0727	2	Lmx1b	1.9847
3	Gm11549	−2.0023	3	Irx3	1.9747
4	Gm21887	−1.9500	4	Foxb1	1.8529
5	Wdr86	−1.7565	5	Irx2	1.8506

### 3.6 GO and KEGG Analysis of DEGs

**FIGURE 5:**
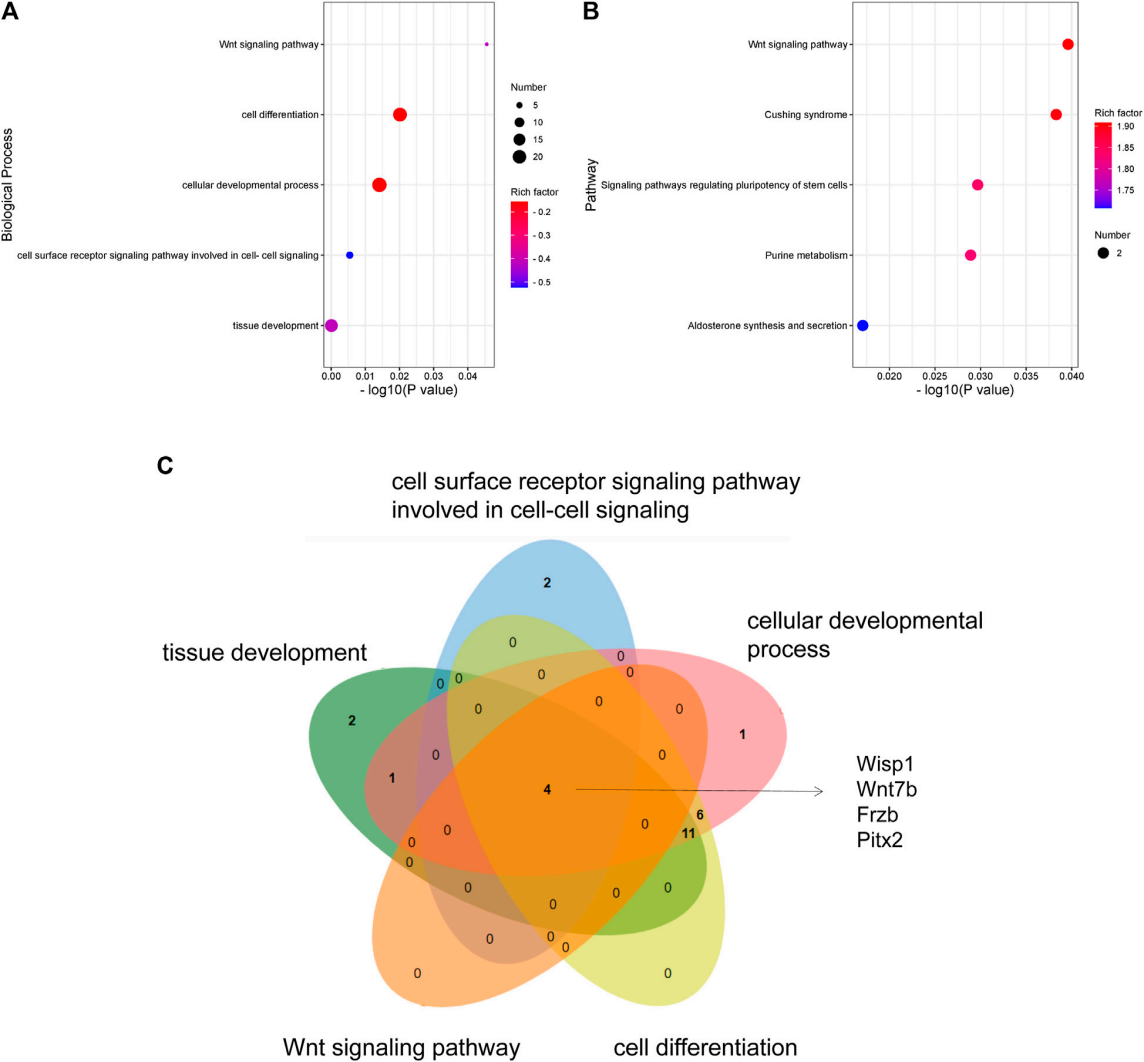
GO and KEGG analyses of DEGs: **(A)** GO enrichment of DEGs. **(B)** KEGG pathway enrichment of DEGs. **(C)** Venn diagram showing the overlapping significant DEGs from GO terms.

To explore the functions of DEGs, GO and KEGG pathway analyses were performed. The GO enrichment analysis showed that 292 GO terms were significantly enriched with the overlapping significant DEGs. As shown in Table 3 and Figure 5A, the significantly enriched GO terms were tissue development, cell surface receptor signaling pathway, cellular developmental process, cell differentiation, and wnt signaling pathway. The intersection DEGs of the aforementioned five pathways in biological processes were Wisp1, Wnt7b, Frzb, and Pitx2 (Figure 5C). The most significant KEGG pathways (*p* < 0.05) related to the overlapping significant DEGs were aldosterone synthesis and secretion, purine metabolism, signaling pathways regulating pluripotency of stem cells, Cushing syndrome, and the wnt signaling pathway (Table 3 and Figure 5B).

**TABLE 3:** GO and KEGG analyses of differentially expressed genes among the sham treatment group, intracerebral hemorrhage model group, and febuxostat group.

GO/KEGG analysis	Description	*p* value	Gene name
GO analysis (biological process)	Tissue development	0.00006	Wnt7b|Wisp1|Lhx1|Klf10|Frzb|Uncx|Lmx1b|Nrtn|St14|Arc|Satb2|Irx3os|Pitx2|Foxb1|Emx1|Irx1|Irx3|Irx2
	Cell surface receptor signaling pathway involved in cell–cell signaling	0.00541	Wisp1|Wnt7b|Frzb|Glra1|Pitx2|Gm12236
	Cellular developmental process	0.01410	Wisp1|Wnt7b|Uncx|Arhgap15|Emx1|Top2a|Lhx1|Klf10|Lhx5|Frzb|Lmx1b|Irx3os|Pitx2|Nr4a2|Tbr1|Foxb1|Nrtn|Barhl2|St14|Arc|Satb2|Irx5|Irx3
	Cell differentiation	0.02013	Wnt7b||Wisp1|Lhx1|Klf10|Lhx5|Pitx2|Frzb|Barhl2Uncx|Tbr1|Lmx1b|Nrtn|Nr4a2|St14|Satb2|Irx3os|Irx5|Foxb1|Emx1|Irx3|Top2a
	Wnt signaling pathway	0.04555	Wisp1|Wnt7b|Frzb|Pitx2
KEGG analysis (pathway)	Aldosterone synthesis and secretion	0.01708	Nr4a2|Agt
	Purine metabolism	0.02891	Rrm2|Gucy2g
	Signaling pathways regulating pluripotency of stem cells	0.02969	Wnt7b|Lhx5
	Cushing syndrome	0.03830	Wnt7b|Agt
	Wnt signaling pathway	0.03961	Wisp1|Wnt7b

### 3.7 Protein–Protein Interaction Network Analysis

**FIGURE 6:**
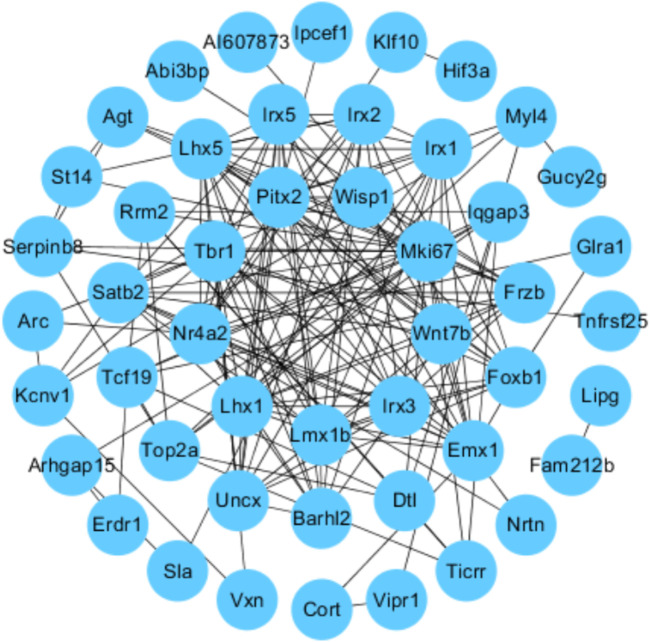
Protein–protein interactions (PPI): STRING analysis with the interaction score set to 0.15. Each node represents the relevant protein, the edge indicates the predicted functional associations, and the number of lines represents the strength of predicted functional interactions between proteins.

We uploaded the overlapping significant DEGs to STRING 11.0 to obtain the PPI network. A total of 49 DEGs were consequently extracted (Figure 6), including Wisp1 and Wnt7b.

### 3.8 Febuxostat Inhibited Wisp1 and Wnt7b Expression After ICH

**FIGURE 7:**
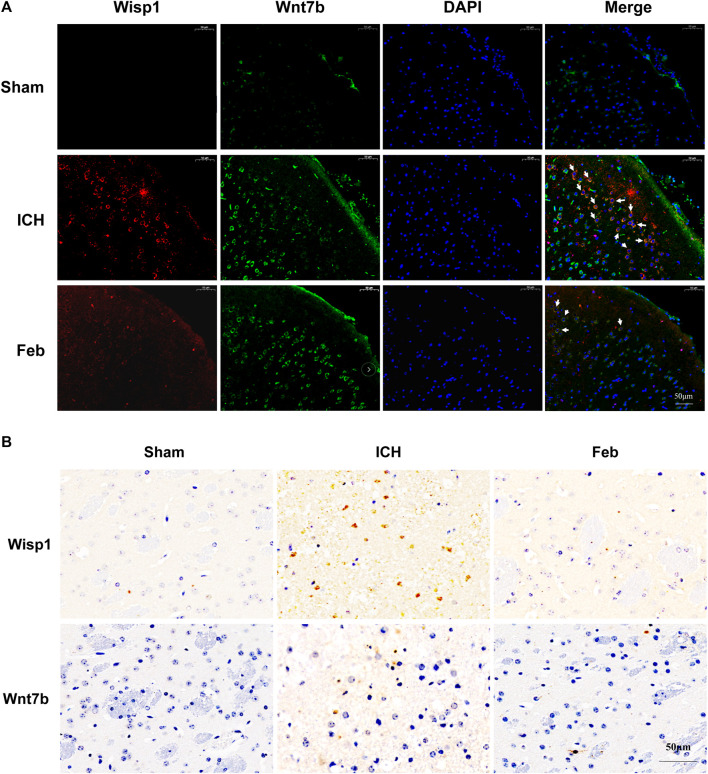
Expression of Wisp1 and Wnt7b in mice: **(A)** Expression of Wisp1 and Wnt7b in the perihematomal area observed by immunofluorescence labeling. Wisp1 is shown in red and Wnt7b is shown in green. White arrows indicate colocalization. Sections were stained with DAPI (blue) to show all nuclei. The scale bar is 50 μm. **(B)** Expression of Wisp1 and Wnt7b in the perihematomal area. The scale bar is 50 μm.

We next examined the expression of Wisp1 and Wnt7b in perihematomal areas by immunofluorescent staining and immunohistochemistry. The results revealed that the expression of Wisp1 and Wnt7b was increased in ICH mice compared with sham mice but was decreased by treatment with febuxostat after ICH (Figures 7A,B).

## 4 Discussion

Febuxostat is an orally administered, nonpurine-selective xanthine oxidase inhibitor being developed for the management of hyperuricemia in patients with gout. The present study revealed the neuroprotective effects of febuxostat and explored its underlying mechanisms.

At present, numerous studies have described the fundamental roles of febuxostat in the improvement of ischemia and reperfusion injury. For instance, febuxostat suppresses ischemia-reperfusion injury of vital organs, such as the kidney and heart (Tsuda et al., 2012; Wang et al., 2015; Khan et al., 2017; Fujii et al., 2019). Febuxostat reduced the induction of endoplasmic reticulum stress, suppressed apoptosis and inflammation by mediating the MAPK signaling pathway, inhibited mitochondrial-dependent apoptosis, and promoted the resynthesis of adenine nucleotides (Tsuda et al., 2012; Wang et al., 2015; Khan et al., 2017; Fujii et al., 2019). However, the role of febuxostat in brain injury after ICH is still unclear. The results showed that febuxostat improved neurological deficits and motor and coordination functions in a mouse model of ICH (Figure 1). Moreover, neuronal cell injury was observed by performing Fluor-Jade B staining and TUNEL staining. The results indicated that febuxostat attenuated neuronal degeneration and apoptosis after ICH (Figure 2). In addition, we also found that febuxostat attenuated BBB permeability after ICH by measuring the extravasation of Evans blue (Figure 3). Febuxostat decreased the levels of IL-1β, IL-6, and IL-18, indicating the inhibition of neuroinflammation after ICH. Taken together, these results suggest that febuxostat protects against brain injury after ICH, probably by alleviating BBB damage and inflammatory injury.

To explore the underlying mechanism of febuxostat in neuroprotection, a whole transcriptome resequencing analysis was performed. In our study, overlapping significant DEGs were identified, and the top five DEGs with log2 (fold change) are shown (Table 2), including the top five downregulated DEGs, namely, Abi3bp, Wisp1, Gm11549, Gm21887, and Wdr86. We further explored the potential functions of the overlapping significant DEGs by GO and KEGG functional enrichment analyses. Among the intersection of DEGs, genes enriched in highly correlated annotation terms were identified. The intersection DEGs of GO terms in biological processes were Wisp1, Wnt7b, Frzb, and Pitx2 (Figure 5C). The most significant KEGG pathways related to cross-talk genes included aldosterone synthesis and secretion, purine metabolism, signaling pathways regulating pluripotency of stem cells, Cushing syndrome, and the wnt signaling pathway (Table 3 and Figure 5B). GO terms and KEGG pathways revealed that DEGs were mainly involved in the wnt signaling pathway. The wnt signaling pathway is one of the most important signaling pathways which regulates cell proliferation, differentiation, apoptosis, stem cell self-renewal, tissue homeostasis, and wound healing. In the wnt signaling pathway, Wnt3a, *β*-catenin, and Frizzled-7 may be associated with cell apoptosis and proliferation and may change the BBB permeability in the ICH model (Zhou et al., 2014; He et al., 2021). Accordingly, we determined the effects of febuxostat on the expression of Wisp1 and Wnt7b (Figure 7). Consistently, the expression of Wisp1 and Wnt7b was increased in perihematomal tissue from ICH mice and was decreased by the administration of febuxostat.

Wnt1-inducible signaling pathway protein 1 (Wisp1) is a cysteine-rich secreted matricellular protein that belongs to the connective tissue growth factor (CTGF) family and is also known as CCN4. Wisp1 is expressed in several tissues, including the epithelium, heart, kidney, lung, pancreas, placenta, ovaries, small intestine, spleen, and brain (Maiese et al., 2012), and it plays an important role in diverse pathophysiological processes, such as embryonic development, inflammation, injury repair, and cancers (Feng and Jia, 2016). It was reported that Wisp1 significantly enhanced the production of pro-inflammatory cytokines (Zhang et al., 2016). Therefore, Wisp1 contributes to ICH in mice and possibly depends on excessive pro-inflammatory cytokine production and inhibition of anti-inflammatory cytokines (Tong et al., 2016; Tong et al., 2018). In our study, Wisp1 was increased in the brain after ICH and was decreased by treatment with febuxostat, suggesting that febuxostat inhibited inflammatory cytokine levels through regulation of Wisp1. In addition, the earlier inflammatory response to ICH includes the activation of local inflammatory factors and the breakdown of the BBB (Askenase and Sansing, 2016). The study also found that febuxostat attenuated BBB damage after ICH. Therefore, febuxostat probably inhibited the production of inflammatory cytokines through the Wisp1-mediated inflammatory response to maintain BBB integrity, thereby protecting against brain injury after ICH. In addition, Wnt7b is a short-range paracrine signal required for programmed cell death by activating the wnt pathway in adjacent vascular endothelial cells (Lobov et al., 2005). Wnt7b, one of the switches driving the proliferative/inflammatory phenotype during cholestasis, could induce the proliferation of cholangiocytes in an autocrine manner and increases the secretion of pro-inflammatory cytokines (Kosar et al., 2021). Thus, Wnt7b induces a proliferative pro-inflammatory program with detrimental effects on disease progression, suggesting that Wnt7b downregulation may contribute to injury. Wnt7b is a wnt ligand and Wisp1 is the target gene of the wnt pathway. However, the relationship between Wnt7b and Wisp1 should be further investigated.

In addition to the wnt pathway, purine metabolism is also one of the pathways enriched by KEGG. On one hand, febuxostat blocked the degradation pathway of adenine nucleotides and promoted ATP recovery to play a protective role in organ injury (Fujii et al., 2019). On the other hand, febuxostat also promoted the recomposition of high-energy phosphates through the blockade of hypoxanthine catabolism (Tani et al., 2019). It indicated that the protective effect of febuxostat on organ injury is related to energy metabolism. Accordingly, the purine metabolism may be one of the signaling pathways regulated by febuxostat in the secondary injury after ICH. Regrettably, the relationship between Wisp1, Wnt7b, and purine metabolism needs to be further studied.

In conclusion, the present study suggested the neuroprotective roles of febuxostat and explored its underlying mechanisms in a mouse model of ICH.

## Data Availability

The data presented in the study are deposited in the GEO repository, accession number GSE193076.
