# Supplementary material for: High-Throughput mRNA Sequencing Reveals Potential Therapeutic Targets of Febuxostat in Secondary Injury After Intracerebral Hemorrhage
Source: Front Pharmacol. 2022 Jun 23;13:833805. doi: 10.3389/fphar.2022.833805 (PMC9260037; doi:10.3389/fphar.2022.833805)
Supplement: Supplementary file 1 [file DataSheet1.docx]

**Supplemental Figure**

| 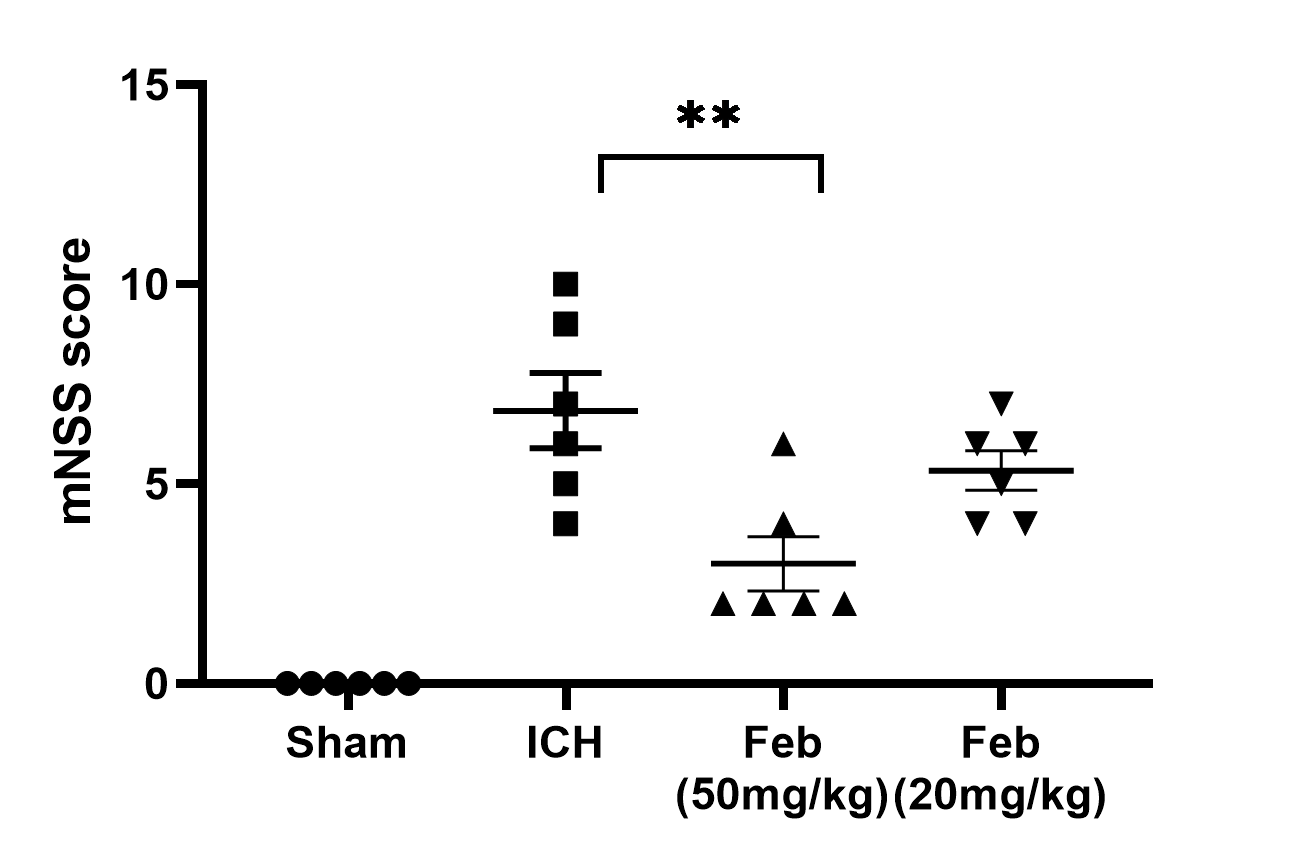 | 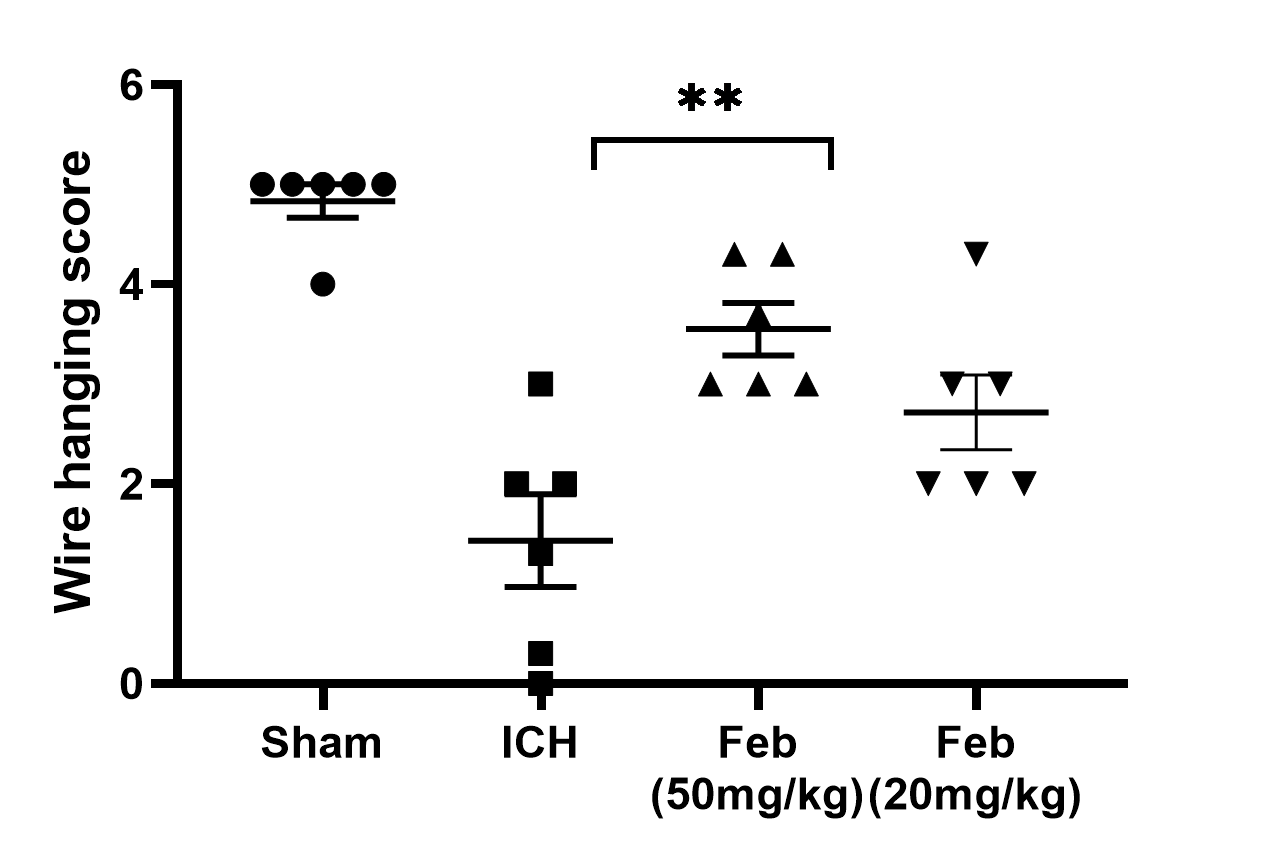 |
| --- | --- |
| (**A**) | (**B**) |
| 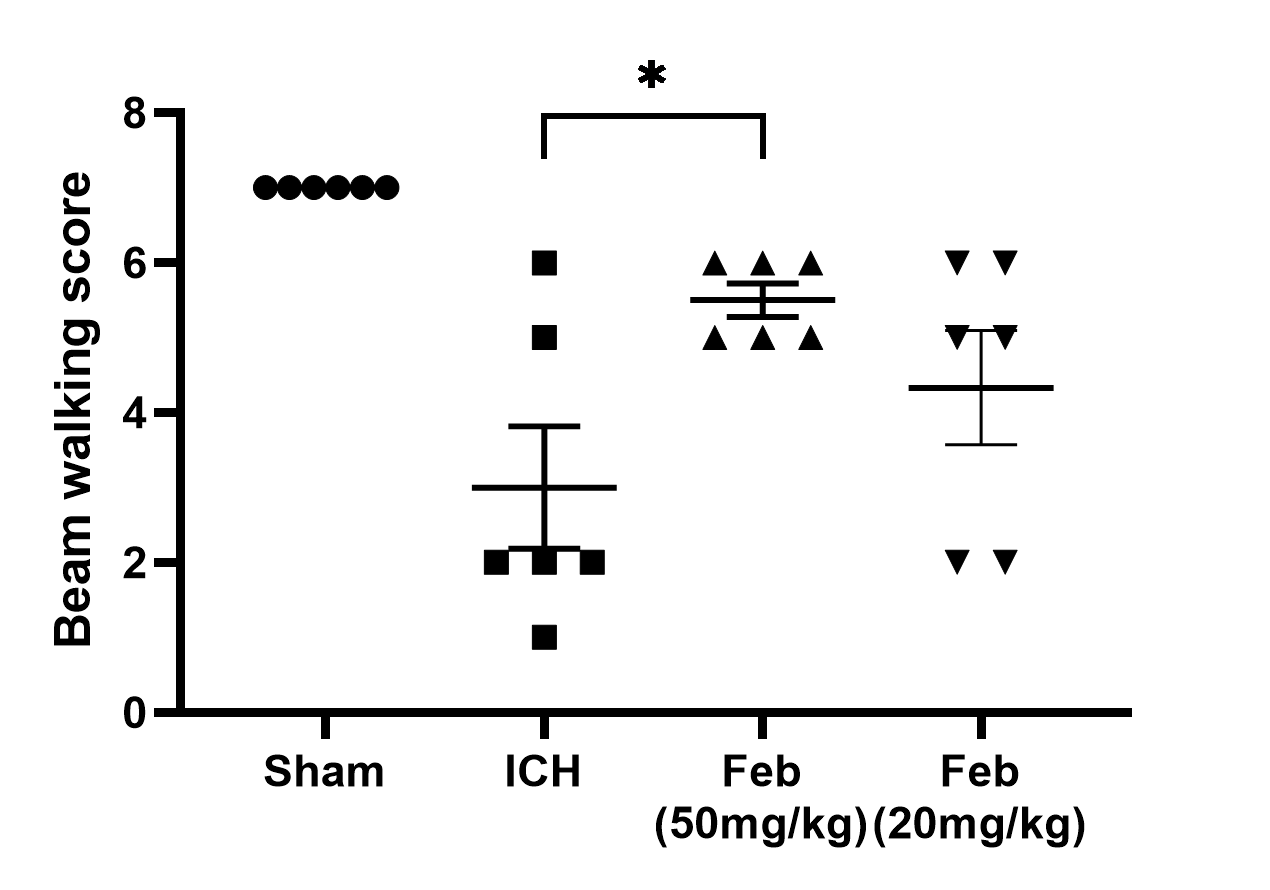 | 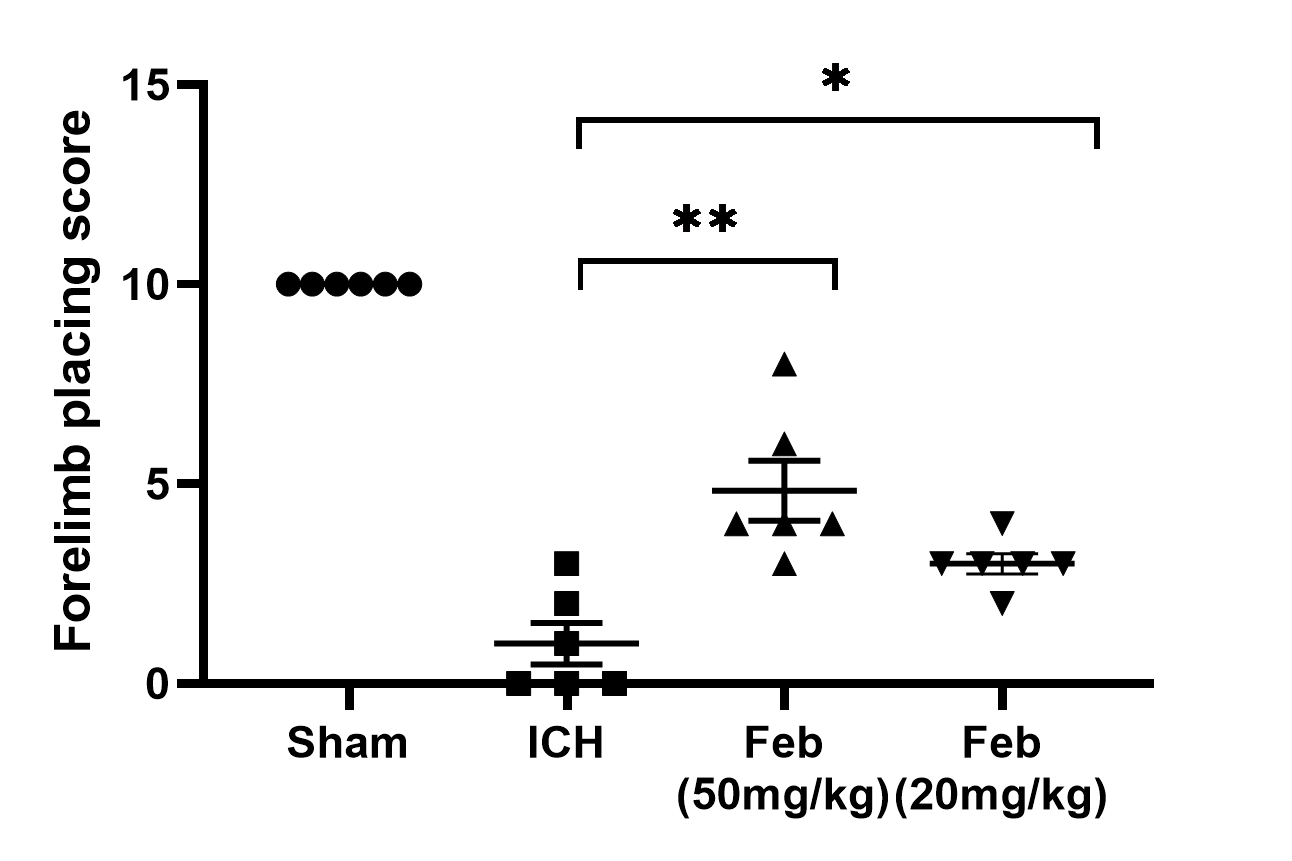 |
| (**C**) | (**D**) |

**Supplemental Figure 1.** Different doses of Febuxostat improved neurological dysfunction after ICH: (A)The modified neurological severity score. (B)The wire hanging test. (C)The beam walking test. (D)The forelimb placing test. Values are mean ± S.E.M; *P < 0.05, **P < 0.01 (n = 6 mice / group, two-way ANOVA)
